# Successful Cardiac Resynchronization Therapy With Defibrillator Implantation in a Patient With Isolated Dextrocardia and Situs Inversus Totalis: Role of High‐Resolution Three‐Dimensional Computed Tomography in Procedural Planning

**DOI:** 10.1002/joa3.70219

**Published:** 2025-11-10

**Authors:** Masako Asami, Yoshinari Enomoto, Naohiko Sahara, Keijiro Nakamura, Hidehiko Hara

**Affiliations:** ^1^ Division of Cardiovascular Medicine Toho University Ohashi Medical Center Tokyo Japan; ^2^ Division of Cardiology National Center for Global Health and Medicine Tokyo Japan

**Keywords:** computed tomography, CRT implantation, dextrocardia

## Abstract

Preprocedural 3D‐CT identified a suitable posterolateral coronary vein in a patient with dextrocardia and situs inversus. Right‐sided CRT‐D implantation guided by 3D imaging achieved optimal lead positioning and biventricular pacing, underscoring the importance of preprocedural anatomical planning in complex congenital settings.

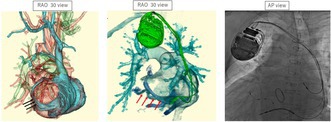

Dextrocardia with situs inversus totalis is a rare congenital anomaly characterized by mirror‐image reversal of thoracic and abdominal viscera, with an estimated incidence ranging from 0.8 to 2 per 10 000 live births [1]. Although the majority of patients with dextrocardia and situs inversus exhibit structurally normal hearts, a subset presents with associated congenital cardiac malformations or venous anomalies, which may complicate interventional procedures. Cardiac device implantation, particularly cardiac resynchronization therapy (CRT), remains technically challenging. Several strategies have been proposed to facilitate CRT device implantation in patients with dextrocardia [2, 3, 4]. However, comprehensive anatomical assessment using high‐resolution, electrocardiogram‐gated, three‐dimensional computed tomography (3D‐CT) has emerged as a promising tool to optimize procedural planning, yet remains underreported in this specific population.

A 60‐year‐old man with ischemic cardiomyopathy, situs inversus totalis, and isolated dextrocardia was referred for CRT‐D implantation. He had a history of anterior myocardial infarction involving the left main coronary artery, treated with percutaneous coronary intervention, followed by surgical mitral valve repair for severe regurgitation. Despite optimal medical therapy, he experienced recurrent heart failure admissions and sustained ventricular tachycardia. On transthoracic echocardiography, left ventricular ejection fraction (LVEF) was 15% with left ventricular dilatation. Mirror‐image electrocardiography (ECG) revealed atrial fibrillation and QRS duration of 142 ms with intraventricular conduction delay (Figure 1A).

**FIGURE 1:**
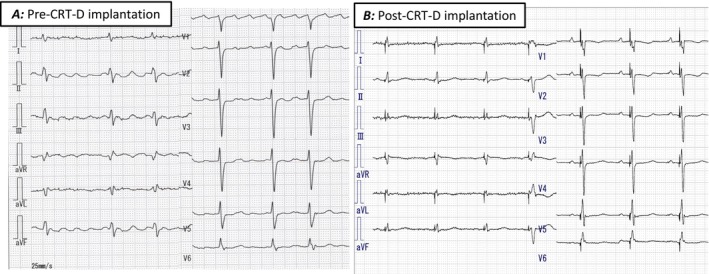
Panel A shows a mirror‐image 12 leads‐ECG on admission which revealed atrial fibrillation and a QRS duration of 143 ms with intraventricular conduction delay. Panel B shows mirror‐image 12 leads‐ECG post CRT‐D implantation which revealed atrial sensing with bi‐ventricular pacing fused optimized intrinsic atrioventricular conduction (QRS duration 124 ms).

Preprocedural high‐resolution, ECG‐gated 320‐row 3D‐CT, performed 2 days prior to the procedure, confirmed dextrocardia, situs inversus totalis, patency of the right subclavian vein, and a suitable lateral branch of the coronary sinus (CS) for left ventricular (LV) lead placement (Figure 2A–C). Contrast‐enhanced imaging was initiated approximately 30 s after intravenous infusion of 70 mL of contrast medium (iopamidol 370 mg/mL), followed by a 20 mL saline flush. To optimize visualization of the coronary venous (CV) anatomy, a region of interest (ROI) was placed at the CS ostium to trigger image acquisition when the CV system was clearly opacified. During the procedure, a right axillary vein approach was used, and fluoroscopic images were horizontally inverted to simulate levocardia. After right‐sided pocket creation, a defibrillation lead was placed in the right ventricular apex. Despite challenges due to right atrial enlargement, CS cannulation was achieved using a wide‐curve guiding catheter. Coronary venography revealed a favorable posterolateral branch consistent with CT findings (Figure 3A,B), and a quadripolar LV lead (Quartet; Abbott, Sylmar, CA) was successfully positioned. Finally, successful cardioversion restored sinus rhythm following atrial lead placement. The postprocedural CT was performed on the following day using the same protocol to confirm lead stability (Figure 2D–F). Device programming with fused optimized intrinsic atrioventricular conduction (SyncAV plus delta value—20%) and interventricular delays (LV proceeding 10 ms at LV 1–2) achieved QRS narrowing from 143 to 124 ms (Figure 1B).

**FIGURE 2:**
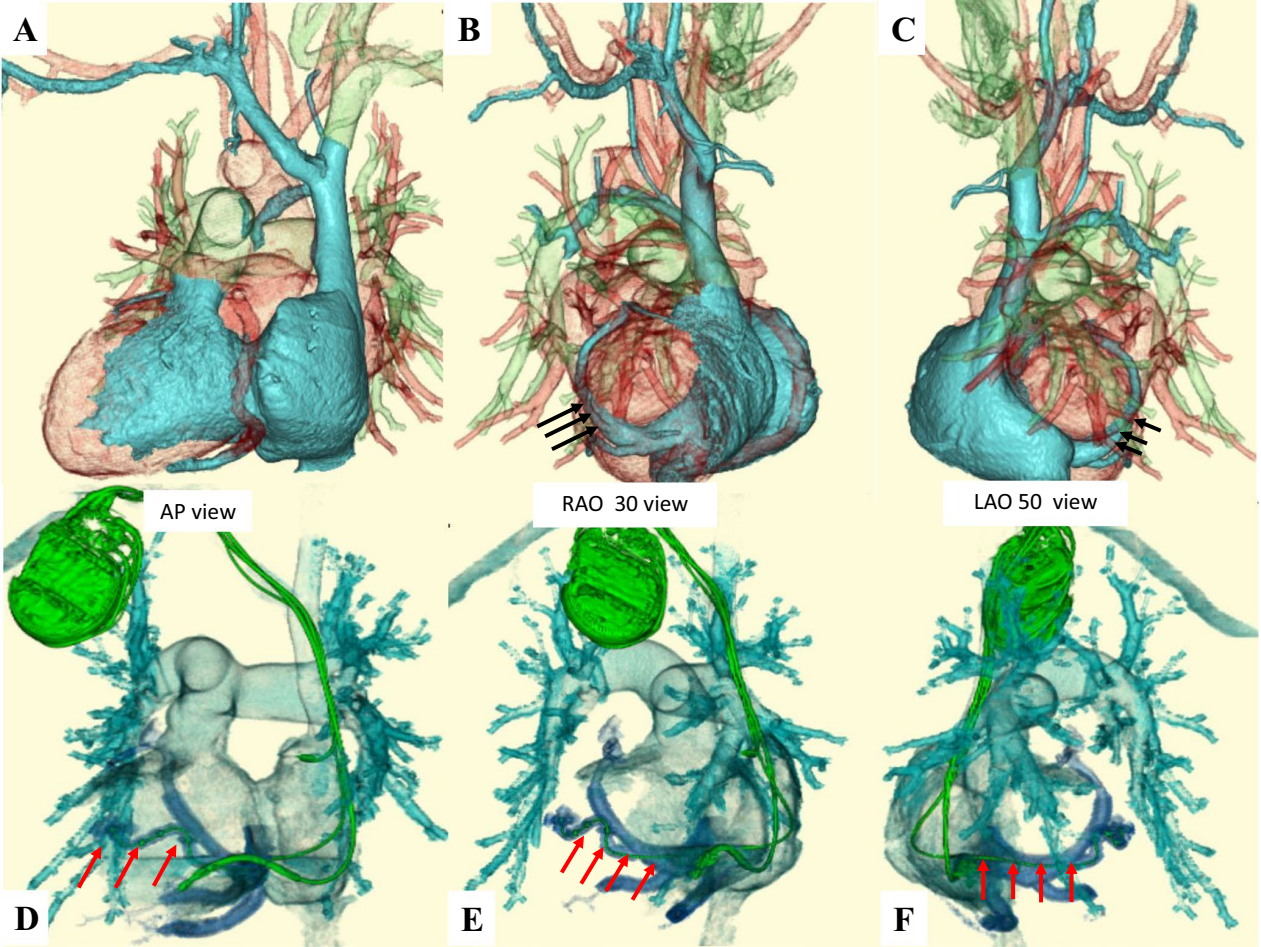
Pre‐procedural (A–C) and post‐procedural 3D‐CT images (D–F). Coronary venous anatomy demonstrates the CS ostium, great cardiac vein (GCV), middle cardiac vein (MCV), and postero‐lateral branches arising from the GCV. The black arrow indicates the targeted vein identified on the pre‐procedural CT, while the red arrow indicates the vein in which the LV lead was actually implanted.

**FIGURE 3:**
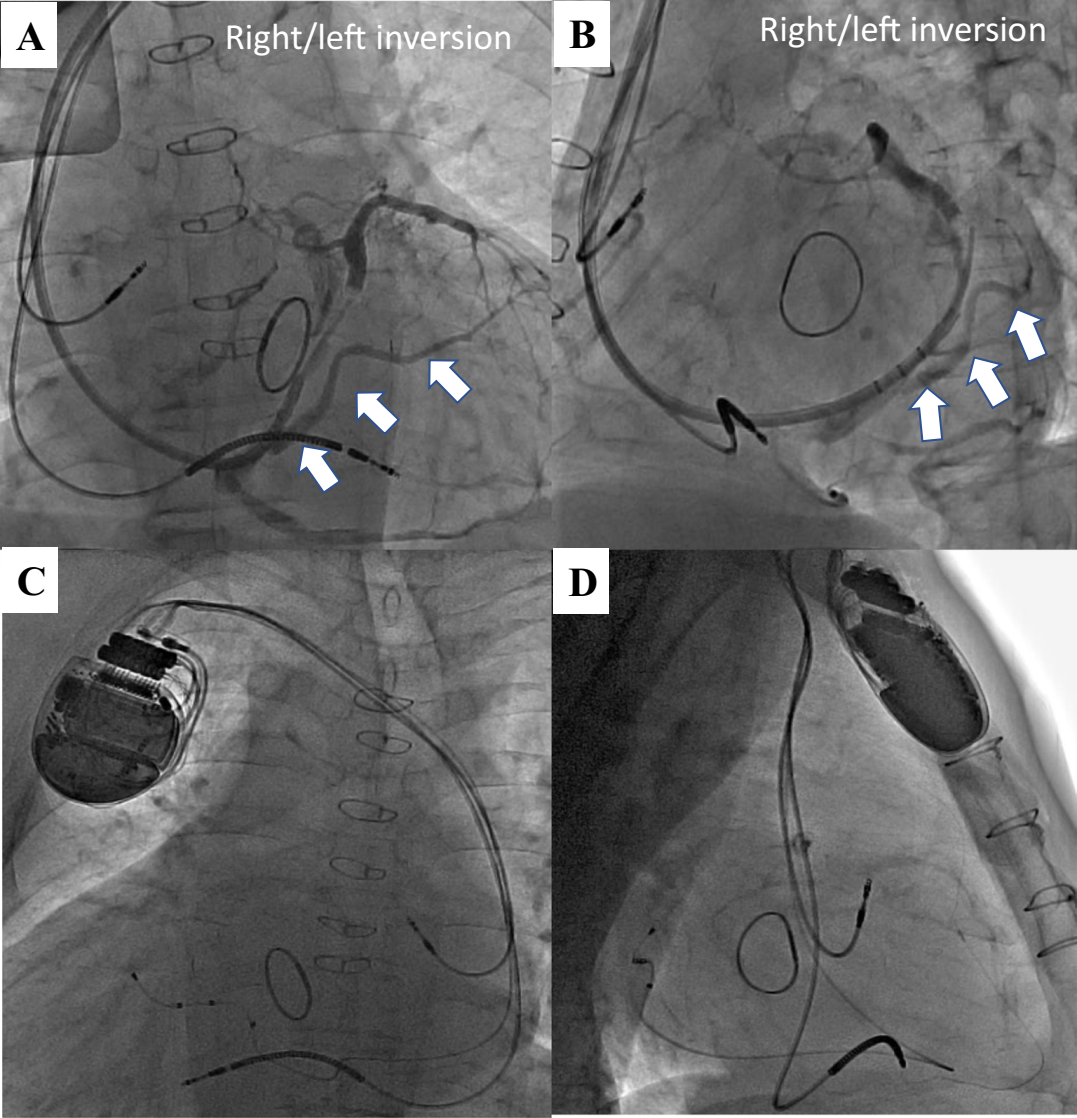
Right and left inverted coronary sinus angiography (A: AP, B: RAO) and the fluoroscopic images after the device implantation (C: AP, D: RL). The white arrows indicate the target branches for the left ventricular lead placement.

We report a successful case of CRT‐D implantation in a patient with dextrocardia and post mitral valve surgery, where the procedure was facilitated by the procedural 3D‐CT images. A previous report described 3D‐CT as a method for preprocedural CRT planning in patients with cardiac malformations [3]. However, this report seems to have only used 3D‐CT to get a rough idea of the anatomical information to determine the feasibility of CRT. We used a 320‐row CT system with an intravenous contrast agent and 0.5‐mm slices under ECG synchronization. Because of the high resolution of the images, we were able to visualize the distal branches of the CS. This image could be freely rotated to visualize the detailed information of the CS branching angle and position, which was useful for planning the catheter and wire manipulation. The 3D‐CT was also imaged to confirm the lead position after the implantation, and the lead position was successfully depicted in detail. A procedural imaging modality is the key to determining a successful implantation in patients with a complex cardiac anatomy. Previous reports of CRT‐D in dextrocardia have relied largely on conventional CT or venography. In contrast, our case demonstrates that high‐resolution ECG‐gated 3D‐CT can directly influence procedural strategy, particularly in the presence of prior cardiac surgery. High‐resolution 3D‐CT can provide critical anatomical insights that facilitate strategic planning and safe, efficient CRT‐D implantation. This imaging‐guided approach should be considered when anatomical complexity or prior surgical intervention is anticipated.

## Conflicts of Interest

The authors declare no conflicts of interest.
